# Surgery for Gastric Remnant Cancer Results in Similar Overall Survival Rates Compared with Primary Gastric Cancer: A Propensity Score-Matched Analysis

**DOI:** 10.1245/s10434-020-08669-2

**Published:** 2020-06-02

**Authors:** Christian Galata, Ulrich Ronellenfitsch, Christel Weiß, Susanne Blank, Christoph Reißfelder, Julia Hardt

**Affiliations:** 1 Department of Surgery, Universitätsmedizin Mannheim, Medical Faculty Mannheim, Heidelberg University, Mannheim, Germany; 2 Department of Visceral, Vascular and Endocrine Surgery, University Hospital Halle (Saale), Martin-Luther-University Halle-Wittenberg, Halle (Saale), Germany; 3 Department of Medical Statistics and Biomathematics, Medical Faculty Mannheim, Heidelberg University, Mannheim, Germany

## Abstract

**Background:**

The purpose of this study was to investigate clinical features, prognostic factors, and overall survival (OS) in surgical patients with gastric remnant cancer (GRC).

**Methods:**

A retrospective analysis of patients with gastrectomy for pT1–4 gastric cancer between October 1972 and February 2014 at our institution was performed. Clinical characteristics were compared between patients with GRC and those with primary gastric cancer (PGC). Multivariable Cox regression analysis was performed to determine the prognostic factors for OS in patients with GRC. A propensity score-matched cohort was used to investigate OS between the GRC and PGC groups.

**Results:**

Of a baseline cohort of 1440 patients, 95 patients with GRC were identified. Patients with GRC underwent more multivisceral resections (*p* < 0.001) than patients with PGC despite lower tumor stages (*p* = 0.018); however, R0 resection rates were not significantly different (*p* = 0.211). The postoperative overall (*p* = 0.032) and major surgical (*p* = 0.021) complication rates and the 30-day (*p* = 0.003) and in-hospital (*p* = 0.008) mortality rates were higher in patients with GRC. In multivariable analysis, the only prognostic factors for worse OS in GRC were higher tumor stage (*p* < 0.001) and the occurrence of postoperative complications (*p* < 0.001). OS between propensity score-matched GRC and PGC groups was not significantly different (*p* = 0.772).

**Conclusions:**

GRC required more invasive surgery than PGC; however, the feasibility of R0 resection was similar. The prognostic factors of GRC were similar to those of PGC, and OS was not significantly different between both groups. Patients with GRC benefit from extensive surgery when performed with low morbidity and mortality.

Gastric remnant cancer (GRC) is defined as an adenocarcinoma arising in the residual stomach after partial gastrectomy and is considered a distinct clinical entity.^1^ Earlier definitions referred only to cancers diagnosed after partial gastric resection for benign indications.^2^ Recently, the definition has changed, and several studies have investigated GRC following oncologic resection for primary gastric cancer (PGC).^3^,^4^ While the overall incidence of PGC is declining worldwide, GRC is currently expected in approximately 1–7% of all patients operated for gastric cancer over the next years and thus remains an important clinical issue.^5^^–^8 Such assumption is based on the high rate of partial gastrectomies performed for peptic ulcer disease until the 1980s, which is before effective medical therapy was widely established, and on the latency period of up to 30–45 years until GRC arises after a previous resection.^9^,^10^ Additionally, a higher GRC incidence after partial gastrectomy for PGC may be expected because of improved oncologic therapy, thereby leading to an increasing number of long-term PGC survivors.^11^ GRC treatment remains a clinical challenge as resection is technically demanding and curative resection rates are low, which is associated with a relatively high incidence of adverse outcomes and poor survival.^12^^–^14 Most previous studies investigated only a small number of patients and compared largely uneven group sizes when analyzing differences in risk factors and patient outcomes between GRC and PGC.^7^,^15^ Therefore, the purpose of this study was twofold: (1) to identify the clinical characteristics and prognostic factors for overall survival (OS) in a large single-center cohort of patients with GRC and PGC, and (2) to compare the OS between GRC and PGC groups using a propensity score matched approach.

## Methods

### Ethics Approval

Ethics board approval was obtained from Medical Ethics Commission II of the Medical Faculty Mannheim, Heidelberg University, Mannheim, Germany (2019-849R). This study was performed according to the Declaration of Helsinki. All patient data used in this analysis were completely anonymized. No direct contact with patients or treating physicians was made for the purpose of the present study.

### Patients

A retrospective analysis of our institutional database for surgical patients with gastroesophageal malignancies was performed. Medical records of 2252 consecutive patients operated between October 1972 and February 2014 were reviewed. Patients who underwent gastrectomy for pT1–4 adenocarcinoma of the stomach were identified. Patients with Barrett’s carcinoma and esophageal cancer were excluded. We also excluded cancers of the esophagogastric junction (Siewert types I and II), because these are classified and staged according to the esophageal scheme in the current American Joint Committee on Cancer/Union for International Cancer Control (AJCC/UICC) system.^16^ A baseline cohort of 1440 patients met the inclusion criteria, of which 95 patients with GRC were identified. GRC was defined as an adenocarcinoma arising in the remnant stomach following a gastric resection for a benign or malignant disease.

### Clinical Characteristics

Data on clinical features and patient outcomes were extracted from the database. For patients with gastric cancer operated between 1972 and 2001, information on AJCC/UICC staging according to the 5th edition of the AJCC/UICC classification was available. The 6th and 7th editions of the AJCC/UICC classification were used from 2002 to 2009 and from 2010 to 2014, respectively. Before the analysis, all patients in this study were restaged according to the 6th edition of the AJCC/UICC staging system for gastric cancer.

Major surgical complications were defined as one of the following events during the postoperative course: anastomotic leak (including duodenal stump blowout), postoperative abdominal abscess, fascial dehiscence, peritonitis, sepsis, secondary hemorrhage, and relaparotomy for any reason. When multiple complications occurred, the most severe complication was used. Complication-related postoperative mortality was presented as postoperative 30-day and in-hospital mortality. Follow-up in the database was based on medical records and direct contact with the patients or with the treating physicians. OS in the database was defined as the interval from surgery to death by any cause or the latest point in time the patient was known to be alive.

### Statistical Analysis

Mean and standard deviation were calculated for quantitative variables. Qualitative variables were quoted as absolute numbers and relative frequencies. The median, with the range or interquartile range, was presented for skewed or ordinally scaled parameters. All statistical tests for the comparison of two groups were two-tailed. Mann–Whitney *U* test was used for continuous variables that were not normally distributed. For qualitative variables, *Χ*^*2*^ test or Fisher’s exact test was used, as appropriate. A test result was considered statistically significant if *p* < 0.05. Univariable and multivariable Cox regression analyses were performed to identify prognostic factors for OS. Variables with significance on the *α* = 0.10 level in the univariable Cox regression analyses were used as covariates in the multivariable Cox regression analysis. In the multiple analysis, backward stepwise selection based on the probability of the Wald statistic was used, and a significance level of *α* = 0.05 was chosen to detect several parameters that might influence the outcome. Hazard ratios were presented together with their 95% confidence intervals (CI). The Kaplan–Meier method was used to estimate survival curves and the log-rank test to compare survival times between groups.

#### Propensity Score Matching

Due to an inhomogeneous distribution of baseline characteristics and uneven group sizes between GRC and PGC, propensity score matching was used to compare OS between GRC and PGC groups.^17^ Only patients with documented follow-up were eligible for propensity score matching. Briefly, a propensity score was calculated using a logistic regression model, in which the type of gastric cancer (GRC or PGC) was regressed as a dependent variable on relevant baseline parameters. The following variables were included in the propensity score to achieve covariate balance of known confounders or known prognostic factors for OS: patient age; year of surgery; AJCC/UICC stage; pT, pN, and M categories; R status; type of gastrectomy; multivisceral resection; signet ring cell carcinoma; and overall and major surgical complications. Patients in the GRC and PGC groups were matched on the logit of the estimated propensity score (1:1 fixed ratio matching) using calipers width equal to 0.05 of the standard deviation of the logit. For clinical considerations, exact matching of R status and M category between the groups was enforced (“exact” method). For all other variables in the model, the “optimal” method option was used. A total absolute propensity score difference of 0.30 for measured covariates suggested an appropriate balance between the groups. Statistical analyses were performed using the SAS statistical analysis software with the PSMATCH procedure (SAS release 9.4, Cary, NC).

## Results

### Patients’ Characteristics

**Fig. 1:**
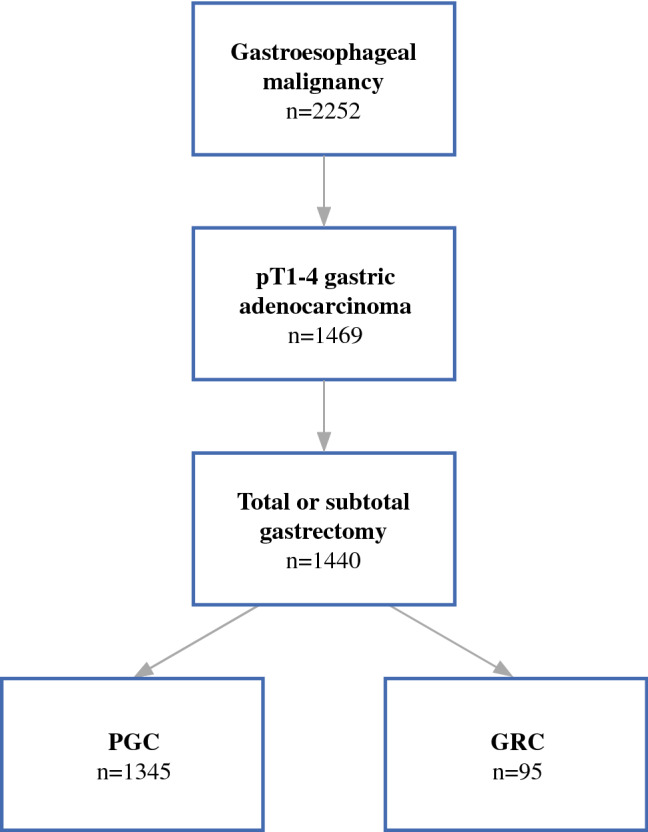
Flow chart of study population selection

A flow chart of the study population is shown in Fig. 1. Of the 1440 consecutive patients who underwent gastrectomy for pT1–4 gastric cancer between October 1972 and February 2014 at our institution, a total of 95 patients (6.6%) with GRC were identified. During the study period, our standard approach for radical resection of gastric cancer was open total or subtotal (4/5) gastrectomy with D2 lymphadenectomy (LAD). As a rigorous standard at our institution, all oncologic resections with curative intent were performed by senior surgeons specialized in upper gastrointestinal surgery.

The vast majority of patients with GRC (86.3%) were male (male-to-female ratio 6.3:1), compared with 54.8% in the unmatched PGC group (*n* = 1345, *p* < 0.001). Specific clinical characteristics of GRC are shown in Table 1. The median age at the time of surgery for GRC was 67 (61–71) years, and the median time interval between initial gastrectomy and surgery for GRC was 25 (14–35) years. For GRC patients with initial gastrectomy for benign disease, the median latency period until development of cancer in the gastric remnant was 30 (range 20–36) years compared with 3 (2–9) years for patients with initial gastrectomy for malignant disease (*p* < 0.001). The median age at the time of initial gastrectomy was 40 (range 32–49) years for all patients with GRC, but 38 (range 30–47) years for patients with initial gastrectomy for benign disease compared with 55 (45–65) years for patients with initial gastrectomy for malignant disease (*p* < 0.001). In almost all cases of GRC, a Billroth II reconstruction was performed after initial gastrectomy (95.7%), which was conducted for benign indications in 82.6% of the patients, and for malignant disease in 17.6% of the patients. Generally, complete resection of the remnant stomach was performed (92.6%).

**Table 1:** Specific clinical characteristics of GRC

Variable	*n* or median	% or IQR
Year of GRC surgery	1982	1978–1988
Type of initial gastrectomy		
Billroth II	89	95.7
Other resections	4	4.3
Histology of initial gastrectomy		
Peptic ulcer	65	81.3
Adenocarcinoma	13	16.3
Other benign	1	1.3
Other malign	1	1.3
Interval (year^a^)	25	14–35
After benign disease	30	20–36
After malignant disease	3	2–9
Age at initial gastrectomy (year)	40	32–49
Benign indication	38	30–47
Malignant indication	55	45–65
Type of GRC gastrectomy		
Total	88	92.6
Subtotal	7	7.4

*GRC* gastric remnant cancer, *IQR* interquartile range

^a^Time interval between initial gastrectomy and surgery for GRC

### Postoperative Outcomes of Patients with GRC and PGC

Table 2 shows the postoperative outcomes of patients with GRC. Median length of hospital stay was 15 (range 13–21) days. The overall complication rate was 37.9%; the major postoperative complication rate 20.0%. Median follow-up time was 23 (range 10–54) months, with a median OS of 29 (95% confidence interval [CI] 11.1–46.9) months.

**Table 2:** Outcomes after surgery for GRC

Variable	*n* or mean	% or IQR
Postoperative morbidity		
Overall complications	36	37.9
Major surgical complications	19	20.0
Early postoperative mortality		
30-day mortality	12	12.6
In-hospital mortality	13	13.7
Length of stay (days)	15	13–21
Follow-up (mo)	23	10–54
5-year survival rate		36.4

*GRC* gastric remnant cancer, *IQR* interquartile range

Comparisons of baseline characteristics between GRC and PGC groups within the unmatched cohort (*n* = 1440) are shown in Table 3. Patients with GRC had higher overall (*p* = 0.032) and major surgical (*p* = 0.021) complication rates and higher postoperative 30-day mortality (*n*_GRC_ = 12, 12.6% vs. *n*_PGC_ = 63, 4.7%; *p* = 0.003) and in-hospital mortality (*n*_GRC_ = 13, 13.7% vs. *n*_PGC_ = 80, 6.0%; p = 0.008) rates than patients with PGC. The rate of total gastrectomies was higher in patients with GRC (*p* < 0.001). Multivisceral resections were performed in 71.6% of the patients with GRC compared with 46.0% of the patients with PGC (*p* < 0.001). The rates of splenectomies (68.4% vs. 32.6%, *p* < 0.001), intestinal resections (9.5% vs. 3.8%, *p* = 0.014), and pancreatic procedures (9.5% vs. 2.9%, *p* = 0.003) were significantly higher in the GRC group compared with the PGC group, whereas the rate of cholecystectomies was significantly lower (5.3% vs. 12.1%, *p* = 0.046). Despite more invasive surgical procedures, patients with GRC showed significantly lower AJCC/UICC (*p* = 0.018) and pN (*p* = 0.001) stages. Based on the 6^th^ edition of the AJCC/UICC staging system on gastric cancer, 50.6% of the patients with GRC had stage I, 25.3% had stage II, 14.8% had stage III, and 9.5% had stage IV disease. Adenocarcinomas of the diffuse type according to Laurén’s classification were diagnosed in 38.7% of the patients with GRC compared with 41.3% in the PGC group (*p* = 0.449). Tumor-free resection margins (R0 resection) were achieved in 86.3% of the GRC cases, whereas 13.7% of the patients had positive resection margins.

**Table 3:** Baseline characteristics of patients with GRC and PGC before and after propensity score matching

Variable	Unmatched cohort (*n* = 1440)					Matched cohort (*n* = 166)				
	GRC		PGC		*p* value	GRC		PGC		*p* value
	*n* or median	% or range	*n* or median	% or range		*n* or median	% or range	*n* or median	% or range	
Age (year)	67	43	65	75	0.184	67	43	65	45	0.343
Year of surgery	1982	40	1986	42	**0.003**	1981	40	1985	39	**0.002**
AJCC/UICC stage					**0.018**					0.123
IA/B	48	50.5	540	40.8		42	50.6	35	42.2	
II	24	25.3	290	21.9		23	27.7	17	20.5	
IIIA/B	14	14.7	310	23.4		11	13.3	24	28.9	
IV	9	9.5	185	14.0		7	8.4	7	8.4	
pT category					0.362					0.183
T1	15	15.8	286	21.3		11	13.3	8	9.6	
T2	55	57.9	679	50.5		50	60.2	44	53.0	
T3	15	15.8	297	22.1		14	16.9	21	25.3	
T4	10	10.5	83	6.2		8	9.6	10	12.0	
pN category					**0.001**					0.485
N0	56	58.9	587	43.7		50	60.2	46	55.4	
N1	28	29.5	456	34.0		26	31.3	28	33.7	
N2	11	11.6	247	18.4		7	0.4	9	10.8	
N3	0	0	53	3.9		0	0	0	0	
M category					0.060					1.000
M0	93	97.9	1225	92.5		81	97.6	81	97.6	
M1	2	2.1	100	7.5		2	2.4	2	2.4	
Resection margins					0.211					1.000
R0	82	86.3	1183	90.4		71	85.5	71	85.5	
R+	13	13.7	126	9.6		12	14.5	12	14.5	
Type of gastrectomy					< **0.001**					1.000
Total	88	92.6	639	47.5		76	91.6	77	92.8	
Subtotal	7	7.4	706	52.5		7	8.4	6	7.2	
Multivisceral resection	68	71.6	611	46.0	< **0.001**	59	71.1	54	65.1	0.506
Signet ring cell carcinoma	17	17.9	365	27.1	0.054	12	14.5	22	26.5	0.083
Postoperative morbidity										
Overall complications	36	37.9	361	27.1	**0.032**	31	37.3	26	31.3	0.513
Major surgical complications	19	20.0	153	11.5	**0.021**	16	19.3	16	19.3	1.000

*p* values in bold type indicate statistical significance

*AJCC* American Joint Committee on Cancer; *GRC* gastric remnant cancer; *PGC* primary gastric cancer; *UICC* Union for International Cancer Control

### Prognostic Factors for OS in Patients with GRC

Univariable and multivariable Cox regression analyses were performed to identify factors that could influence OS in GRC (Table 4). In the univariable analysis, higher AJCC/UICC stage (*p* < 0.001), higher pT (*p* = 0.001), and pN (*p* < 0.001) categories, and the occurrence of postoperative complications (*p* < 0.001) were significantly associated with worse OS. In the multivariable analysis, only higher AJCC/UICC stage (*p* < 0.001) and overall complications (*p* < 0.001) remained as risk factors for decreased OS in patients with GRC.

**Table 4:** Prognostic factors for OS in patients with GRC based on univariable and multivariable Cox regression analyses

Variable	Univariable *p* value	Multivariable *p* value	Hazard ratio (95% CI)
Gender	0.504	–	–
Age	0.597	–	–
Year of GRC surgery	0.602	–	–
Type of initial gastrectomy (BII vs. other resections)	0.263	–	–
Histology of initial gastrectomy (benign vs. malign)	0.561	–	–
Time interval (initial gastrectomy to surgery for GRC)	0.964	–	–
Type of GRC gastrectomy (total vs. subtotal)	0.457	–	–
Multivisceral resection	0.424	–	–
Laurén’s type (diffuse vs. non-diffuse)	0.270	–	–
Signet ring cell carcinoma	0.573	–	–
AJCC/UICC stage	< **0.001**	< **0.001**	**1.615 (1.285–2.029)**
pT category	**0.001**	–	–
pN category	< **0.001**	–	–
M category	0.287	–	–
Resection margins (R+ vs. R0)	**0.001**	–	–
Overall complications	< **0.001**	< **0.001**	**2.911 (1.616–5.243)**
Major surgical complications	**0.009**	–	–

*AJCC* American Joint Committee on Cancer, *CI* confidence interval, *GRC* gastric remnant cancer, *OS* overall survival, *UICC* Union for International Cancer Control

*p* values in bold type indicate statistical significance

### OS Between GRC and PGC in a Propensity Score-Matched Cohort

**Fig. 2:**
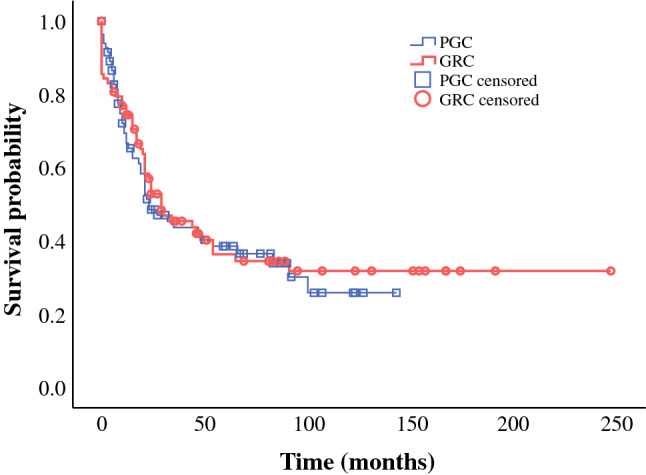
Kaplan–Meier survival curves. No significant difference in OS was observed between the propensity score-matched GRC and PGC groups (*p* = 0.722). *GRC* gastric remnant cancer, *OS* overall survival, *PGC* primary gastric cancer

In the unmatched cohort (*n* = 1440), multiple clinically relevant baseline characteristics were significantly different between patients with GRC and those with PGC (Table 3). To adjust for potential confounders, OS between both groups was compared using a propensity score-matched cohort (*n* = 166) using 1:1 matching of 83 patients with GRC with follow-up data to 83 patients with PGC. A total absolute propensity score difference of 0.30 for measured covariates suggested an appropriate balance between the two groups. All matched patients in the GRC and PGC groups were different individuals. Comparison of the baseline characteristics between the matched groups revealed no significant differences, except for year of surgery where a 4-year difference in the median year of operation (1981, range 40 years vs. 1985, range 39 years) was statistically significant (*p* = 0.002). In the matched PGC group, the vast majority of tumors (86.8%) were located proximally. Figure 2 shows the Kaplan–Meier survival curves of propensity score matched GRC and PGC groups. Comparison of OS between both groups revealed no statistically significant difference (*p* = 0.772). The estimated 5-year survival rates of the matched GRC and PGC patients were 36.4% and 38.6%, respectively.

## Discussion

We presented data on 95 consecutive patients with GRC from a cohort of 1440 patients with pT1–4 gastric adenocarcinoma who underwent gastrectomy over a 42-year period. Our analysis is one of the largest single-center cohorts of patients with GRC in comparison to the cohort sizes in previous studies.^7^,^10^ We found that surgery for GRC resulted in similar oncologic outcomes compared to surgery for PGC. This finding is of particular importance as it suggests that patients with GRC may have a good and realistic chance of long-term survival if they undergo radical resection. In these patients, surgery may be technically demanding, which explains the higher rates of surgical complications and complication-related early postoperative mortality observed in our study. Therefore, surgery for GRC should be conducted in designated surgical centers with the aim to deliver state-of-the-art, high-quality treatment with low morbidity and mortality to ensure the best possible oncologic outcomes. The observed mortality rate of 13.7% in patients with GRC might seem excessively high. The fact that the median year of surgery for GRC patients was 1982 certainly explains some of this high mortality. Surgical technique and perioperative care were less advanced in earlier times, which resulted in a higher incidence of complications to start with, and in a higher probability for failure to rescue (own unpublished data). Moreover, patients with GRC in our cohort underwent a high rate of multivisceral resections, and we also included patients with positive resection margins and metastatic disease. These factors also may have contributed to a higher mortality rate.

GRC was identified in 6.6% of the baseline cohort, which is consistent with the observation in the literature where approximately 1–7% of all patients operated for gastric cancer have been reported to have GRC.^5^^–^8 In our study, patients with GRC showed distinct clinical features. Moreover, we observed a higher proportion of men than women (ratio 6.3:1) and Billroth II resections were mostly performed as the preceding operations (95.7%).^7^,^10^,^13^ The overwhelming majority of the patients in the GRC group underwent completion total gastrectomy as total resection of the gastric remnant is regarded as the standard approach for this disease.^10^ Multivisceral resections, including a high rate of splenectomies, were more common in GRC compared to PGC, which is consistent with the findings of Ohashi et al., who recommend total gastrectomy with splenectomy for GRC after distal gastrectomy for gastric cancer.^3^ In addition to more extensive resections, surgery for GRC is likely to be more invasive due to adhesions caused by the previous procedures, especially when those were performed for malignant disease where surgery includes lymph node dissection, omentectomy, or bursectomy.^18^ This greater extent of surgical trauma may have potentially contributed to the significantly higher rates of postoperative morbidity and mortality in the GRC group in our study. Efforts have been made to diagnose GRC at an early stage, and close endoscopic surveillance of patients undergoing partial gastrectomy has been recommended, especially after Billroth II resections and for long time intervals.^19^ Although data on how the patients with GRC in our study were followed up after initial resection of the stomach were not available, endoscopic surveillance could explain the significantly lower pN and AJCC/UICC stages in the GRC group.

In this study, the R0 resection rate for GRC was 86.3%, which coincides with the R0 resection rates in the literature (77–85%).^7^,^20^,^21^ Notably, the R0 resection rate was not significantly different between the GRC and PGC groups in the unmatched cohort, suggesting that despite the potentially more demanding surgical conditions in GRC, the feasibility of radical resection is similar to that in patients with PGC.

GRC is regarded as a distinct clinical entity.^1^ In this study, we also reported on the different clinical characteristics between GRC and PGC groups. However, when we analyzed the prognostic factors for OS in patients with GRC, univariable and multivariable analyses revealed that the risk factors for decreased OS were similar to those established for PGC.^22^^–^24 Only advanced AJCC/UICC stage and the occurrence of postoperative complications were independent prognostic factors in multivariable analysis. Other studies identified similar risk factors for decreased OS in GRC.^3^ When OS was compared between GRC and PGC groups in a propensity matched cohort, no statistically significant differences were detected. Thus, our data suggest that while GRC may be different from PGC regarding surgical strategy and postoperative morbidity, GRC is comparable to PGC in terms of prognostic factors and OS. This finding may seem surprising as GRC has been associated with low rates of curative resection and poor survival.^12^^–^14 However, a closer look at the literature also revealed that although several authors indicated poor prognosis for GRC, they did not report that the survival outcomes of GRC were significantly worse than those of PGC within their study cohort. Viste et al. investigated 819 patients with GRC from the Cancer Registry of Norway from 1970 to 1979 and found that the apparent poor prognosis for GRC relative to PGC is attributed to differences in patient and tumor characteristics.^13^ Other retrospective analyses involving single centers also failed to reveal differences in OS when comparing GRC and PGC.^7^,^21^

Given the long latency period from initial gastrectomy to the development of gastric remnant cancer, it seems irritating that there is no tangible age difference at diagnosis between patients with GRC and PGC. However, when looking at subgroups of patients with GRC, it becomes obvious that the latency period is much longer in patients with precedent benign gastric disorders than in patients who had previously been operated on for malignant neoplasms. In turn, the former group of patients underwent the initial gastrectomy at a much younger age than the latter, which resulted in a similar age at the time of completion gastrectomy. This finding implies that the risk for GRC following gastrectomy for benign indications persists over a very long time, whereas following gastrectomy for malignancies, it is highest in the first couple of years.

We used a propensity score matched model to compare the OS between GRC and PGC. As the incidence of GRC is considerably lower than that of PGC, other studies have compared survival using largely uneven group sizes. Two highly cited relevant publications compared survival data of 50 patients with GRC to those of 516 patients with PGC, and the data of 52 patients with GRC to those of 656 patients with PGC.^7^,^20^ Other studies also had substantial differences in the number of patients between GRC and PGC.^14^,^25^,^26^ When significant differences in clinically relevant baseline characteristics are present in two groups of uneven size, propensity score matching can be used to achieve covariate balance of known confounders and to allow the investigation of patient outcomes adjusted for tumor stage and other potentially interfering parameters.^17^

This study has some limitations. The study design was retrospective, and the study period spanned more than 40 years. Thus, some variables might have been assessed and classified differently over the years, which could potentially introduce bias and limit the validity of the analyses. There may be known or unknown confounders that were not assessed and thus not available for propensity score matching and analysis. Particularly, no continuous documentation of the extent of LAD or the administration of perioperative chemotherapy was available over the long period covered in this study. Because data on the extent of LAD were not available, it is possible that the lower pN and AJCC/UICC stages that we observed in GRC were the result of less radical LAD due to more difficult surgical conditions. This would imply that the patients with GRC were understaged; however, this is unlikely as surgery for GRC was significantly more extensive than the surgery for PGC in our cohort. Furthermore, if patients with GRC had been understaged, one would expect worse OS among these patients, which we did not observe in the matched cohort. Data on neoadjuvant and adjuvant treatment were not documented in our database before the year 2005 and 2007, respectively. As the majority of patients with GRC in our study underwent surgery before these dates, perioperative chemotherapy administration could not be included as a prognostic factor in our analysis. However, as we compared GRC and PGC in a cohort matched for several clinically relevant parameters, such as tumor stage, which also determined the administration of adjuvant therapy, adjuvant treatment is unlikely to be a relevant confounder in this study. In addition, in other studies on GRC adjuvant chemotherapy was not a significant factor for OS, neither in univariable nor in multivariable analyses.^3^

## Conclusions

In this large, single-center analysis, the feasibility of R0 resection in GRC was similar to that in PGC. Although GRC required a more invasive surgery, which is associated with higher rates of early postoperative morbidity and mortality, the OS of propensity score matched GRC and PGC patients was not significantly different. Advanced AJCC/UICC stages and the occurrence of postoperative complications were the only negative prognostic factors for GRC in multivariable analysis. Despite the differences in clinical features, GRC is comparable to PGC in terms of prognostic factors and oncologic outcomes. Patients with GRC benefit from extensive surgery when performed with low morbidity and mortality.
