# Correction to: Surgery for Gastric Remnant Cancer Results in Similar Overall Survival Rates Compared with Primary Gastric Cancer: A Propensity Score-Matched Analysis

**DOI:** 10.1245/s10434-021-10917-y

**Published:** 2021-10-18

**Authors:** Christian Galata, Ulrich Ronellenfitsch, Christel Weiß, Susanne Blank, Christoph Reißfelder, Julia Hardt

**Affiliations:** 1 Department of Surgery, Universitätsmedizin Mannheim, Medical Faculty Mannheim, Heidelberg University, Mannheim, Germany; 2 Department of Visceral, Vascular and Endocrine Surgery, University Hospital Halle (Saale), Martin-Luther-University Halle-Wittenberg, Halle (Saale), Germany; 3 Department of Medical Statistics and Biomathematics, Medical Faculty Mannheim, Heidelberg University, Mannheim, Germany

## Correction to: Ann Surg Oncol (2020) 27:4196–4203 https://doi.org/10.1245/s10434-020-08669-2

The article Surgery for Gastric Remnant Cancer Results in Similar Overall Survival Rates Compared with Primary Gastric Cancer: A Propensity Score-Matched Analysis, written by Galata et al., was originally published electronically on the publisher’s internet portal on June 2, 2020, without open access. With the authors’ decision to opt for Open Choice the copyright of the article changed on September 29, 2021, to ©The Author(s) and the article is forthwith distributed under a Creative Commons Attribution 4.0 International License.

Open Access funding enabled and organized by Projekt DEAL. The original article has been updated.

